# Two-dimensional finite element analysis of elastic adhesive contact of a rough surface

**DOI:** 10.1038/s41598-020-61187-9

**Published:** 2020-03-25

**Authors:** Harish Radhakrishnan, Sreekanth Akarapu

**Affiliations:** ANSYS Inc., 2600 ANSYS Drive, Canonsburg, PA 15317 USA

**Keywords:** Mechanical engineering, Computational science

## Abstract

Adhesive contact of a rigid flat surface with an elastic substrate having Weierstrass surface profile is numerically analyzed using the finite element method. In this work, we investigate the relationship between load and contact area spanning the limits of non-adhesive normal contact to adhesive contact for various substrate material properties, surface energy and roughness parameters. In the limit of non-adhesive normal contact, our results are consistent with published work. For the adhesive contact problem, we employ Lennard-Jones type local contact interaction model with numerical regularization to study the transition from partial to full contact including jump-to-contact instabilities as well as load-depth hysteresis. We have investigated evolution of bonded contact area and pull-off force for various surface roughness parameters, substrate material properties and surface energy. We have identified two non-dimensional parameters to adequately explain experimentally observed adhesion weakening and strengthening phenomena. A design chart of the relative pull-off force as function of non-dimensional parameters is also presented.

## Introduction

Smooth surfaces when brought into close proximity spontaneously jump into contact and require a finite force to pull them apart. This finite pull-off force quantifies adhesion or adhesive strength of the bond between two solids. The weakness or lack of adhesion in most of real-world phenomena is attributed to surface contamination and lack of proximity between surfaces, which is measured on the scale of the range of van der Waals interactions. Surface roughness, which precludes surfaces from coming into close contact, is observed to be the dominant factor in comparison with surface contamination contributing to the loss of adhesion^1^.

Based on ASME B46.1, even the finest surface finish for industrial applications is about 10–15 times the range of interaction *α*~1.0 *nm*. In automotive industry applications, the surface finish ranges from about 75–1250 *α*. These high ratios of roughness to range of interaction is one of the reasons for adhesion to be a less critical factor in design considerations of industrial machine components. On the other hand, in applications such as sensors and actuators, MEMS devices have high surface to volume ratio with length scales ranging from micron to nanoscale. The reliability of assembly and operation of these devices is observed to primarily depend on adhesion^2–4^. The goal of this work is to contribute to the quantification of adhesion in terms of non-dimensional parameters towards developing an engineering design chart.

The loss of adhesion with an increase in roughness is observed in several experiments^5–11^. These experiments can be grouped based on a nondimensional parameter $\beta =(\gamma /{E}^{\ast }\alpha )$, the ratio of surface energy density γ to product of modulus E^∗^ and range of interaction *α*. Quon *et al*.^7^ investigated the contact between rough gold on a smooth mica surface which is on the lower end on $\beta  \sim 2\times {10}^{-3}$ scale and authors found that there is more than ~80% reduction in adhesion for an increase in roughness by ~1–1.5 *α*. Gui *et al*.^8^ investigated the effect of roughness on bondability of Si wafers, which has $\beta  \sim 7\times {10}^{-4}$, and reported that spontaneous bonding transitions to loss of bonding for an increase in roughness from 0.1 to 1.0 *α* Fuller and Tabor^9^ studied the adhesion of soft rubbers on rough perspex surface with *β* ~ 50–500 and showed a decrease in the rate of loss of adhesion with increase in roughness for more compliant rubber. Fuller and Roberts^10^ performed rolling experiments of soft rubber on a rough surface with *β* ~ 30–1500 and showed both loss and gain of adhesion with increase in roughness. For *β* ~> 250, in the low roughness regime, adhesion is observed to increase with roughness until a critical value followed by loss of adhesion. Guduru^11^ performed experiments using even softer rubberlike material with $\beta  \sim {10}^{4}$ on a rigid wavy surface and found similar adhesion strengthening with increase in roughness and attributed it to the contact instabilities and hysteresis in loading and unloading.

The seminal work by Fuller and Tabor^9^ was one of first comprehensive efforts in developing a model to explain the experimental results of adhesion loss due to increase in roughness. In this work, they have used Johnson-Kendell_Roberts (JKR)^12^ approximation of adhesion between elastic spheres in combination with Greenwood-Williamson (GW)^13^ statistical multi-asperity contact model to explain loss of adhesion using a dimensionless parameter $\theta ={E}^{\ast }{\sigma }^{3/2}/{R}^{1/2}\gamma$ where *σ*, *R* being root mean square (RMS) roughness and radius of curvature respectively. Maugis^14^ and Morrow^15^ used a similar approach to investigate DMT^16^ approximation and the transition from JKR and DMT respectively using multi-asperity contact models for rough surfaces. In all the above-mentioned studies, the adhesive contact was at the individual asperity scale and the asperities are non-interacting. These models cannot capture the important observed phenomena such as the jump-to-contact instabilities and hysteresis in loading and unloading due to surface roughness. In contrast, the work done in references^17–23^ have studied contact instabilities during loading and unloading of adhesive micro-contact. Komvopoulos^24^ used constitutive relations between interfacial force and separation at the asperity level exhibiting jump-in instability in conjunction with GW multi-asperity model and introduced a new adhesion parameter which is the ratio of rms roughness to equilibrium interatomic distance.

The traditional GW multi-asperity surface model does not include the long-range elastic interactions of asperities which leads to qualitatively and quantitatively different contact morphologies. Persson^25^ developed a contact mechanics theory for self-affine fractal surfaces, which approximately includes elastic interactions. Persson’s theory explained the importance of including power spectrum of heights, long-range elastic interactions and showed linearity between load-contact area and fractal nature of the contact morphology. In the non-adhesive limit, Hyun *et al*.^26^ performed a comprehensive numerical study of contact between a rigid flat surface and an elastic substrate with self-affine fractal rough surface and showed that the range of linearity of load and contact area, contact morphology and pressure distribution are quantitatively different from the predictions of GW multi-asperity type contact models. These differences in contact morphology due to long-range elastic interactions will have a significant influence on the adhesive contact behavior of rough surfaces. Although the aforementioned references are relevant and seminal works, they are by no means exhaustive and the authors refer to most elegant and comprehensive review on the topic by Ciavarella *et al*.^27^.

In the present work, we study the contact between a flat rigid surface and an elastic substrate with rough surface characterized by a Weierstrass function. The interaction is modeled using Lennard-Jones interaction potential with a viscous regularization to handle the jump to contact instabilities. The adhesive contact is solved in ANSYS, a general-purpose finite element solver, with nonlinear large deformation effects. We have investigated the contact area evolution and pull-off force for various RMS roughness, RMS slope, Hurst exponent, substrate modulus and found two non-dimensional parameters to explain both adhesion strengthening and weakening observed in experiments.

## Results

### Evolution of Contact Area

Real contact area has significant engineering importance in the thermal management of electrical connections in micro and nano devices. The stiffness of joints in aerospace and microelectronic devices to random vibrations depends largely on the real contact area and its morphology^28^. In this section, we discuss the evolution of contact area in the presence of adhesion and the limiting case of non-adhesive contact.

**Figure 1:**
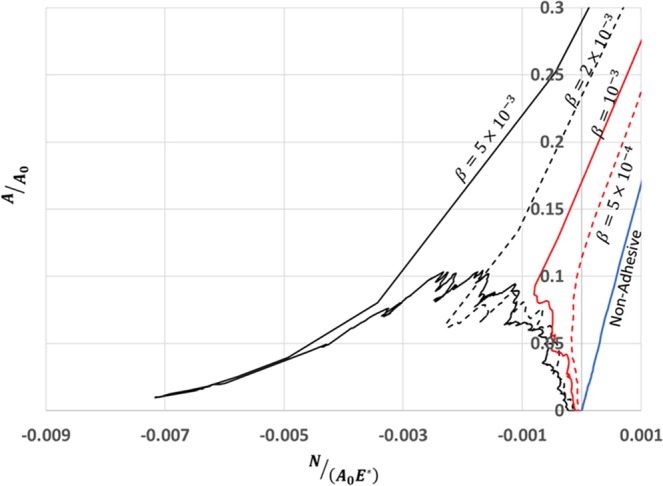
A plot of normalized contact area with normalized load for adhesive contact for various values of *β* in comparison with limiting case of non-adhesive contact for a system size of 8192 with $h{\text{'}}_{rms}=0.0123$, *H* = 0.7.

For different values of *β*, during approach, the evolution of contact area with load is shown in Fig. 1. Unlike the gradual and linear evolution of real contact area *A* with load *N* for non-adhesive contact, the adhesive contact is zero until a net attractive load and suddenly jumps into contact. Moreover, there are several contact instabilities during the development of adhesive contact area. This behavior is similar to the experimental results published in Waters *et al*.^29^.

In the non-adhesive limit, it is well established that the contact area is proportional to applied load with range of linearity from about 5–8% relative contact area^26^. In agreement with published work, the non-dimensional quantity $\kappa =(A{E}^{\ast }{h{\prime} }_{rms}/N)$ is independent of system size and constant within 5–8% relative contact area (see Fig. S4 in supplemental information). For H and *v* equal to 0.5 and 0.3 respectively, the value of *k* is about 2.35 which in agreement with Hyun *et al*.^26^ and lies within that reported by Bush *et al*.^30^ and Persson^31^. Here, ${E}^{\ast }=E/(1-{\upsilon }^{2})$ is the effective modulus, with *E* and *v* being Youngs modulus and poisons ratio. During non-adhesive contact (see Fig. S5 in supplemental information), the evolution of the heights power spectrum clearly shows that the increase in contact area is accompanied by the increase in the length scales over which the contact is accommodated. This contrasts the contact evolution of traditional GW type models which does not include the long-range elastic interactions of the asperities.

**Figure 2:**
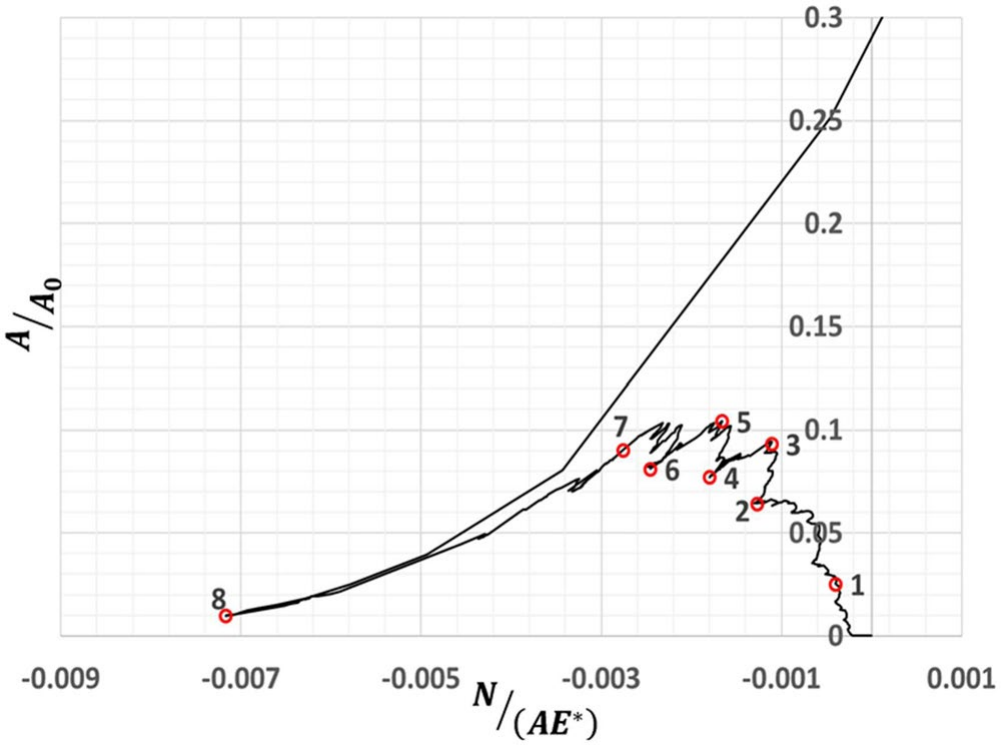
A plot of normalized contact area with normalized load for $\beta =5\times {10}^{-3}$ with red open circles investigated in Figs. 3 and 4 to study the adhesive contact evolution.

In contrast, as shown in Fig. 2, the adhesive contact evolution starts with a jump to contact at a finite net attractive load and increases from state ‘1’ to ‘5’ in a sequence of jump to contact instabilities followed by a decrease in contact area to state ‘8’. To understand the adhesive contact evolution, we have investigated the contact pressure and gap profiles at various states to elucidate the intermittent crack zipping and unzipping as the mechanism of adhesive contact attachment and detachment respectively (see Supplementary Information for further details).

**Figure 3:**
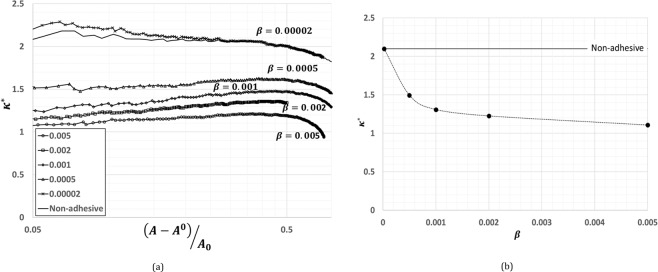
(**a**) A plot between normalized incremental contact area from the bonded state with normalized load for various values of *β*. The plot shows the proportionality at low loads. (**b**) A plot of ${\kappa }^{\ast }=(A-{A}^{0}){E}^{\ast }{h{\prime} }_{rms}/N{A}_{0}$ as a function of *β*. The plot shows that ${\kappa }^{\ast }\to \kappa$ as $\beta \to 0$.

In case of adhesive contact, the contact area at net zero load defined as bonded contact area *A*^0^ is of great engineering importance. As it can be seen from Fig. 1, the bonded contact area *A*^0^ increases with increasing *β*. Similar to non-adhesive contact, we have investigated the relation between load and change in contact area from bonded state. In Fig. 3a, we have plotted a non-dimensional quantity k^*^(see Eq. 1), similar to *k*, as a function of change in contact area about bonded state relative to nominal contact area *A*_0_, $(A-{A}^{0}/{A}_{0})$.

1 $${\kappa }^{\ast }=(A-{A}^{0}){E}^{\ast }{h{\prime} }_{rms/N{A}_{0}}$$

It turns out that k* is constant and independent of *h*_*rms*_ and *h*′_*rms*_. As shown in the Fig. 3b, the value of k* for various values of *β* approaches the value of *k* in the non-adhesive limit. The value of k* is found to decay with increase in *β* as a power law. For an adhesive contact, at constant value of *β*, this relationship can be used to estimate the value of k* and in turn, the bonded contact area.

### Pull-off force and Scaling

**Figure 4:**
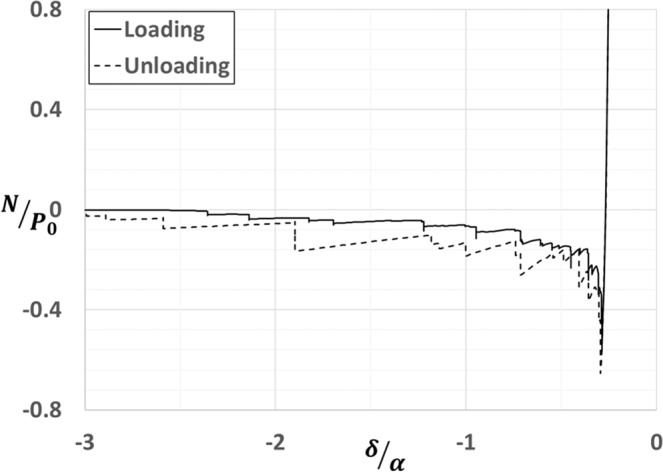
A plot of normalized load vs. average separation during a loading cycle of approach and detachment of adhesive contact. The plot shows hysteresis due to surface roughness.

**Figure 5:**
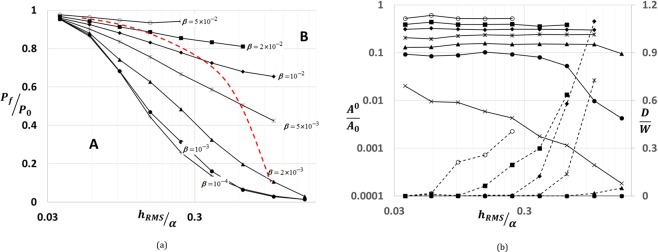
(**a**) A plot of normalized pull-off force as function of normalized roughness for various values of $\beta$. The red dashed line separates the regions with (right of the dashed line) and without (left of dashed line) dissipation/hysteresis. (**b**) A plot of relative bonded contact area ${A}^{0}/{A}_{0}\,$and normalized dissipation $D/W$vs. normalized roughness ${h}_{rms}/\alpha$ for various values of $\beta$. The symbols in the plot take the corresponding values of $\beta$ shown in Fig. 7a. The solid lines and dashed lines correspond to ${A}^{0}/{A}_{0}\,$and $D/W$ respectively.

Besides jump to contact instabilities, an adhesive contact exhibits hysteresis in an approach and detachment cycle. As shown in load-displacement plot (see Fig. 4), our approach can capture both the instabilities and hysteresis during loading and unloading. The maximum load during detachment of contact is defined as the pull-off force. Pull-off force can also be considered as a measure of the strength of adhesion. Similar to the experimental results reported in^9,10^, as shown in Fig. 5a, the relative pull-off force *P*_*f*_/*P*_0_ decays with increasing normalized surface roughness *h*_*rms*_/*α*. Here, ${P}_{f}$ and ${P}_{0}$ are pull-off forces for rough and smooth surfaces respectively. Additionally, at constant ${h{\prime} }_{rms}$, the rate of decay of ${P}_{f}/{P}_{0}\,$decreases with increase in the value of *β*. For low values of *β*, corresponding to stiff solids, the results are in accordance with the experiments on direct wafer bonding^8^.

To understand the effect of hysteresis on pull-off force, we have investigated the ratio of energy dissipated (D) during a cycle to the amount of work done during approach (W) as a function of ${h}_{rms}/\alpha$ (see Fig. 5b). For each value of *β*, the onset of dissipation occurs at different values of roughness. Using these critical values of surface roughness, as shown in Fig. 5a, the red dashed line marks the onset of hysteresis dividing the space in regions A and B. In region B, at constant value of ${h}_{rms}/\alpha$, the increase in pull-off force for a given increase in *β* is larger in comparison to that in region A. This implies that the parameter ${h}_{rms}/\alpha$, acts to weaken and strengthen adhesion in region B and solely has a weakening effect in region A. Additionally, as shown in Fig. 5b, the ratio of bonded to nominal contact area ${A}^{0}/{A}_{0}$ also increases with increase in *β*. For a given value of *β* and ${h{\prime} }_{rms}$, higher the value of ${A}^{0}/{A}_{0}$, lower is the rate of decay of relative pull-off force ${P}_{f}/{P}_{0}$ with ${h}_{rms}/\alpha$. Hence, for a given ${h{\prime} }_{rms}$, relative pull-off force is a function of *β*, ${h}_{rms}/\alpha$ and ${A}^{0}/{A}_{0}$.

**Figure 6:**
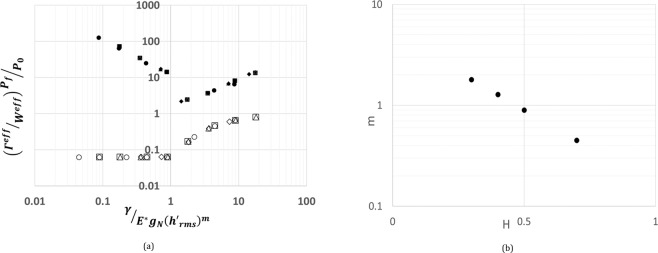
(**a**) A plot of ratio of total surface energy to strain energy (${\Gamma }^{eff}/{W}^{SE}$) at pull-off for various values of slopes computed at constant ${h}_{rms}=0.62$ indicated by closed symbols ⦁0.0325, ▪0.02, ▴0.0123, ◆0.0076 and relative pull-off ${P}_{f}/{P}_{0}$ for various values of slopes indicated by corresponding open symbols with respect to $\gamma /{E}^{\ast }{g}_{N}{({h{\prime} }_{rms})}^{m}$. (**b**) A plot of the exponent *m* having a power law scaling with Hurst exponent.

**Figure 7:**
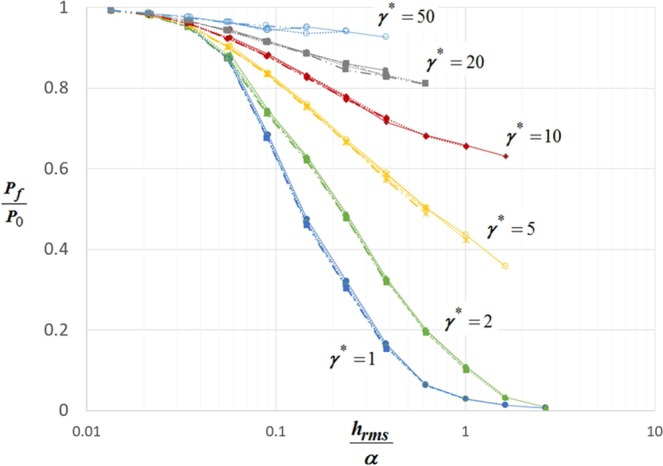
A plot of relative pull-off force as a function of normalized roughness for various values of ${\gamma }^{\ast }=\gamma /{E}^{\ast }{g}_{N}{({h{\prime} }_{rms})}^{m}$.

In Fig. 6a, at a constant ${h}_{rms}/\alpha$, we have investigated the effect of ${h{\prime} }_{rms}$ on relative pull-off force ${P}_{f}/{P}_{0}$. As it can be seen, we could collapse $\,{P}_{f}/{P}_{0}$ data by scaling ${h{\prime} }_{rms}$ as ${\gamma }^{\ast }=(\gamma /{E}^{\ast }{g}_{N}{({h{\prime} }_{rms})}^{m})$. Here g_*N*_ is the small-scale amplitude and the exponent *m* has an exponential decay with Hurst exponent H (see Fig. 6b). In the fractal limit, the quantity ${g}_{N}{({h{\prime} }_{rms})}^{m}$ scales as ${\lambda }_{N}^{H+(H-1)m}\,$ which is convergent for *H* ≥ 0.5. This convergence of ${\gamma }^{\ast }$ is similar to the conclusions found in references^32–38^ and in contrast with work done in refs. ^39,40^. Hence, as shown in Fig. 7, for various values of $\beta$, ${h}_{rms}$, ${h{\prime} }_{rms}\,$and H, $\,{P}_{f}/{P}_{0}$ is a function of two non-dimensional parameters ${\gamma }^{\ast }$ and ${h}_{rms}/\alpha$.

## Discussion

**Figure 8:**
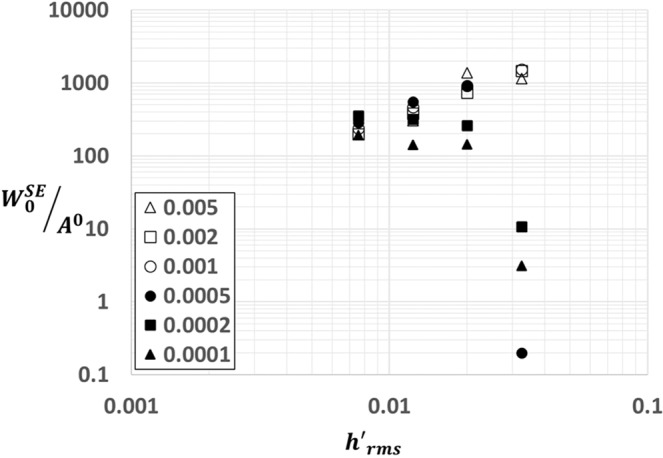
A plot of strain energy at bonded state per unit bonded contact area ${W}_{0}^{SE}/{A}^{0}$ as a function of $h{\text{'}}_{rms}$ for various values of $\beta$. The data in this plot is computed at constant ${h}_{rms}=0.62$.

The strength of adhesive contact of a rough surface is a strong function of the statistical properties of the surface roughness. It is a ubiquitous natural observation that rougher surfaces adhere weakly or not at all compared to the smoother counterparts. Moreover, softer polymers such as glues, pressure sensitive adhesives tend to be sticky on relatively rougher surfaces. These observations suggest an intimate competition between surfaces roughness and material softness towards both strengthening and weakening adhesion. In this study, we have identified two non-dimensional parameters, ${h}_{rms}/\alpha$ and ${\gamma }^{\ast }$, to influence adhesion of rough surface. The two asymptotic limits of this phenomena are the one with weak interactions and stiff solids and the other being strong interactions and soft solids. It is a well established result that surface roughness ${h}_{rms}$ monotonically decreases adhesion close to the limit of weak interactions and stiff solids. But, in the transition towards the softer solids and stronger interactions, ${h}_{rms}$ tends to contribute both to weakening and strengthening of adhesion. This effect is due to increase in energy dissipated due to contact instabilities. Therefore, for a constant ${h{\prime} }_{rms}$, adhesion is solely influenced by ${h}_{rms}/\alpha$. For a given ${h}_{rms}$, especially in the neighborhood of soft solids and strong interactions, strength of adhesion is also influenced by the strain energy stored near the interface at the bonded state. This energy can be interpreted as the available energy to pry the surfaces apart which depends on the contact area and its morphology at the bonded state. As shown in Fig. 8, this energy per unit bonded contact area has a power law scaling with ${h{\prime} }_{rms}$ for cases in the neighborhood of soft solids and strong interactions (higher values of *β*). In the other limit, there is barely any correlation with ${h{\prime} }_{rms}$ which implies that ${h}_{rms}/\alpha$ is the only relevant parameter for low values of $\beta$ and ${\gamma }^{\ast }$influences adhesion for moderate to high values of $\beta$.

**Figure 9:**
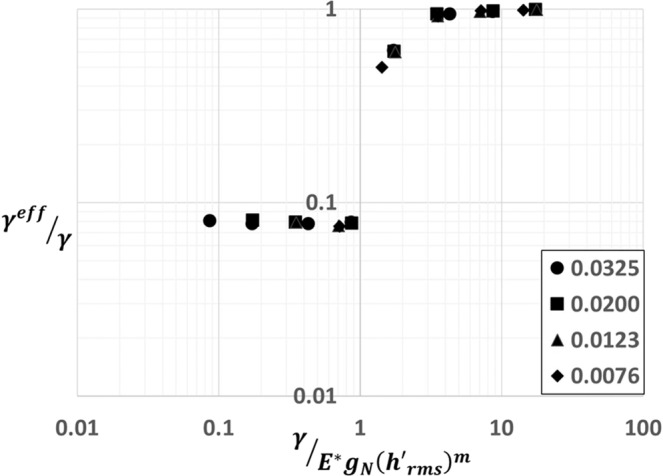
A plot of the ratio of surface energy density at pull-off to surface energy density from LJ potential ${\gamma }^{eff}/\gamma$ with respect to $\gamma /{E}^{\ast }{g}_{N}{({h{\prime} }_{rms})}^{m}$ for various values of slopes. The data in this plot is computed at a constant ${h}_{rms}=0.62$.

For a physical understanding of the parameter ${\gamma }^{\ast }$, following the energetic arguments of Johnson’s parameter^41,42^, we have investigated the relation between ${\gamma }^{\ast }$ and the ratio of surface energy ${\Gamma }^{eff}$ to strain energy ${W}^{SE}$ at pull-off. The surface energy ${\Gamma }^{eff}$ denotes the amount of surface energy gained by the system if the surfaces are separated at pull-off. ${W}^{SE}$ is computed by subtracting the strain energy due to average pressure, homogenous part, from the total strain energy stored in the system at pull-off. As shown in the Fig. 6a, with decreasing ${\gamma }^{\ast }$, rapid increase in ${\Gamma }^{eff}\,/{W}^{SE}$ suggests that ${W}^{SE}$ tends to zero faster than ${\Gamma }^{eff}\,$while approaching the non-adhesive limit. Beyond ${\gamma }^{\ast }$ equal to one, we found a power law scaling between ${\Gamma }^{eff}\,/{W}^{SE}$ and ${\gamma }^{\ast }$. The ratio ${\Gamma }^{eff}\,/{W}^{SE}$ at pull-off when expressed in terms of energy densities as ${\gamma }^{eff}\,/{U}^{SE}{d}^{i}$, length scale ${d}^{i}\,$emerges as the depth over which the inhomogeneous strain energy decays. It turns out that ${d}^{i} \sim {h}_{rms}.$ Here, ${\gamma }^{eff}$ is computed as ${\Gamma }^{eff}\,/{\lambda }_{0}$. We have also investigated ratio ${\gamma }^{eff}\,/\gamma$ in relation to ${\gamma }^{\ast }$ for various values of ${h{\prime} }_{rms}$ as shown in Fig. 9. Similar to relative pull-off force ${P}_{f}/{P}_{0}$, ${\gamma }^{eff}\,/\gamma$ also has a sharp transition beyond ${\gamma }^{\ast }$equal to one. Hence, the condition for an appreciable adhesion can be quantified as ${\gamma }^{\ast } > 1$ for $H\ge 0.5$. To make sense of our criterion, we have looked at Dalhquist’s criterion^36^, an empirical criterion for pressure sensitive adhesives which bounds the modulus of adhesive to about 0.3 MPa for stickiness. This criterion is an industry standard for the design of pressure sensitive adhesive tapes. For polymers, assuming an average chain length on the order of a micron, computing small scale amplitude ${g}_{n}$ along with taking $\gamma  \sim \,$50 mJ/m^2^, $h{\text{'}}_{rms}=0.01$ and $H=0.7$, $E < 0.3\,MPa$ for ${\gamma }^{\ast } > 1$ which agrees very well with Dalhquist’s criterion.

This work is limited to two-dimensions as it is computationally formidable to consider three-dimensional case. Although a deterministic Weierstrass profile is considered, it captures self-affinity of heights observed in real surfaces. We believe that the self-affinity of any rough surface model is the most important characteristic to adequately capture the contact evolution and scaling.

## Conclusions

In this work, we have investigated adhesive contact mechanics of rough surface characterized by a Weierstrass function using a consistent numerical approach in two dimensions. By capturing the contact instabilities and hysteresis, we have elucidated the approach and detachment of an adhesive rough contact to a process akin to interface crack zipping and unzipping. More importantly, we have identified two non-dimensional parameters, ${h}_{rms}/\alpha$ and ${\gamma }^{\ast }$, which influence adhesion and shown that ${\gamma }^{\ast } > 1$ can be considered as stickiness criterion.

## Methods

Real surfaces such as asphalt road, fractured rock and perspex surfaces are generally rough with a defining property of being self-affine fractal over several decades of length scales^43,44^. In this paper, we employ Weierstrass function to model the rough surface profile of two-dimensional elastic substrate. The Weierstrass function is given by

2 $$h(x)={\sum }_{n=0}^{N}{g}_{n}\,\cos (2\pi x/{\lambda }_{n})N\to \infty $$

The height profile is a spatially periodic function with infinitely many length scales. The amplitude ${g}_{n}$ at each length scale is proportional to ${\lambda }_{n}^{H}$, where H is the Hurst exponent (0 < H < 1). The wavelength ${\lambda }_{n}$ is defined as

3 $${\lambda }_{n}={\lambda }_{0}{\xi }^{-n}$$

The large length scale ${\lambda }_{0}$ which is equal to the system size is subdivided into smaller length scales ${\lambda }_{n}$ by integer powers of $\xi$ (see Eq. 3).

As real surfaces have a physical lower length scale cut-off such as lattice constant for crystalline materials and fine grain size in disordered materials such as fractured rock surface, we use two times the equilibrium distance of interaction potential as the smallest length scale. In this work, we restrict the length scales to integers by considering ${\lambda }_{0}$ being powers of 2 with $\xi$ equal to 2 and investigated system sizes from 64 to 16384. We have investigated surfaces with values of H equal to 0.3,0.5 and 0.7.

The general description of the procedure for generating a typical finite element mesh with rough surface profile for various root mean square roughness ${h}_{rms}$ and slope $h{\text{'}}_{rms}$ is described in *Supplementary Information*.

To model the adhesive interaction between the flat rigid surface and elastic substrate with rough profile, we have implemented a local contact law using Lennard-Jones (LJ) type force separation law (see Eq. 4).

4 $$p(\alpha )=(8\gamma /3{\alpha }_{0})({({\alpha }_{0}/\alpha )}^{9}-{({\alpha }_{0}/\alpha )}^{3})$$

The length dimension of our simulations is considered in units of equilibrium distance *α*_0_ of the LJ interaction model. To capture the jump to contact instability during the approach of two surfaces, we have added damping to the LJ force separation law. The added damping to the interaction law simulates the contact behavior as Maxwell type viscoelastic material with nonlinear spring in series with dashpot. The damping parameter and local interaction model was thoroughly validated in the study of adhesion between smooth elastic spheres by comparing against load-depth curves obtained using RIKS arc-length method^45^. In this work, we have restricted the damping induced energy to a minimum in comparison with the strain energy stored in the body.

The boundary value problem of adhesive contact is simulated in general-purpose finite element software ANSYS using large strain formulation under quasi-static indentation. The custom interaction model is implemented through a user subroutine USERINTER.

## Supplementary information

Supplementary Information.

## Data Availability

The data used in this manuscript can be obtained from the authors upon reasonable request.
