# Supplementary material for: Two-dimensional finite element analysis of elastic adhesive contact of a rough surface
Source: Sci Rep. 2020 Mar 25;10:5402. doi: 10.1038/s41598-020-61187-9 (PMC7096536; doi:10.1038/s41598-020-61187-9)
Supplement: Supplementary file 1 — Supplementary Information. [file 41598_2020_61187_MOESM1_ESM.docx]

**Supplemental Information**

**Two-dimensional finite element analysis of elastic adhesive contact of a rough surface**

Harish Radhakrishnan and Sreekanth Akarapu*

ANSYS Inc., 2600 ANSYS Drive, Canonsburg, PA 15317, USA

*Corresponding Author

Senior Application Engineer

15915 Katy Fwy, Suite 550

Houston, TX-77094

Cell: 5095925194

Email: sreekanth.akarapu@gmail.com

Figure below shows a typical mesh used consisting of 4-noded quadrilateral plane strain elements. The mesh close to the contact surface consists of elements with uniform edge size of 1-unit length (${\lambda_{N}}/2$). The total height of this zone is at least ${10h}_{rms}$. The nodes on the contact surface are then moved vertically by *h*(*x*) (see Eq. 2) to create the rough surface profile. To prevent distorted elements, the elements below also have their nodes moved vertically by a fraction of *h*(*x*). The contact surface profiles for these meshes have $h_{rms}^{'}$ constant by choosing appropriate values of $g_{N}$ even when $\lambda_{0}$ range from 64 to 16,384 length units. Further from this zone, the elements are graded with increasing size towards the far field boundary. To ensure that the far field boundaries do not influence the solution, we generate a graded size mesh with an increasing element size. The distance of the boundary from the surface is at least ${1000h}_{rms}$. This ensures that any component of stress measured at the boundary is homogenous. Selected runs performed by doubling and quadrupling this distance confirm the absence of boundary effects as no measurable differences are seen in the load-depth or load-contact area relations.


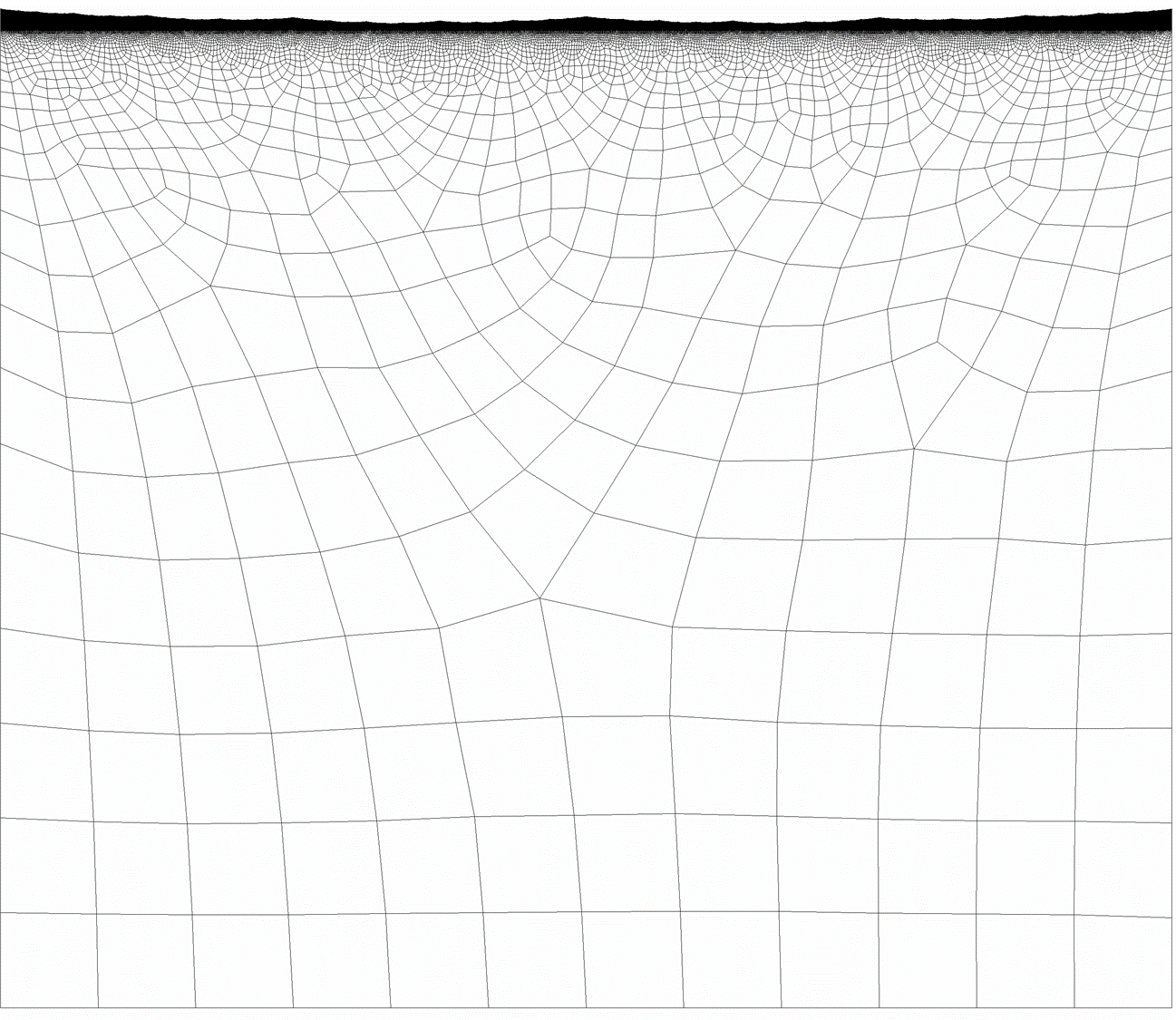


Fig. S1 Typical finite element mesh of the elastic substrate with wierestrass surface profile


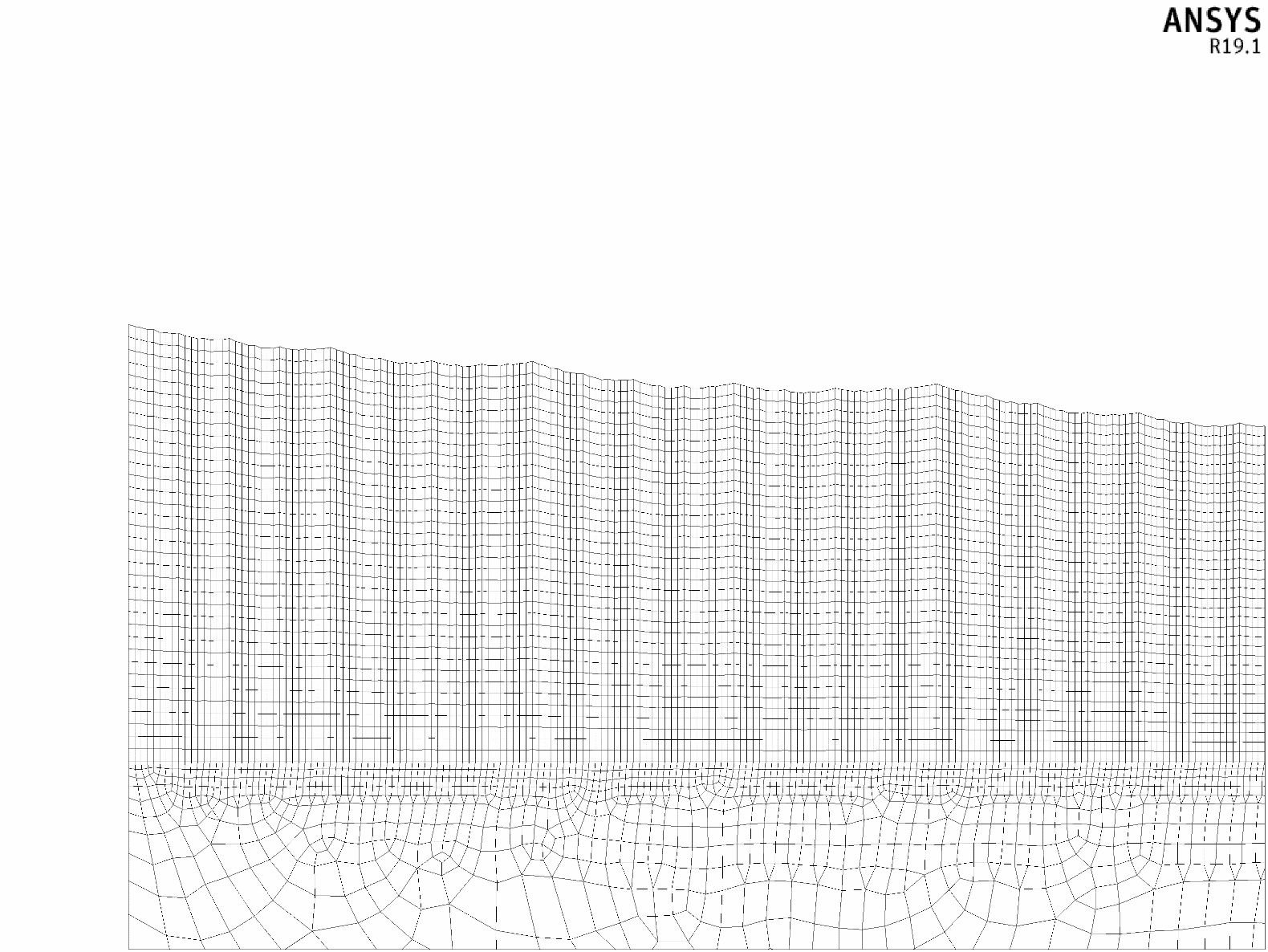


Fig. S2 Exploded view of the mesh near the surface. The vertical dimension is scaled 10 times to show the nature of the rough profile.

**Mesh Convergence Study**

The adequacy of the mesh size at the region close the contact surface (Fig S2) is verified by refining the mesh in this region. The default size of the element 1 length units is refined to 0.5, 0.25 and 0.125 length units. Selected runs are performed for $\lambda_{0}$=2048 using the refined meshes for all values of $\gamma^{*}$ reported in Figure 10. For all the mesh sizes and $\gamma^{*}$ studied here, the magnitude of pull of loads are within 1% of each other. Figure S3 shows the normalized load depth curves for different mesh sizes and $\gamma^{*}$=20. We emphasize the close agreement of the pull off load values between the different mesh sizes. The load depth curves have minor differences due to the subtle changes in the unstable attachment and detachment of asperities which however do not affect the magnitude of the pull of load. The results help validate the adequacy of the mesh sizes used.


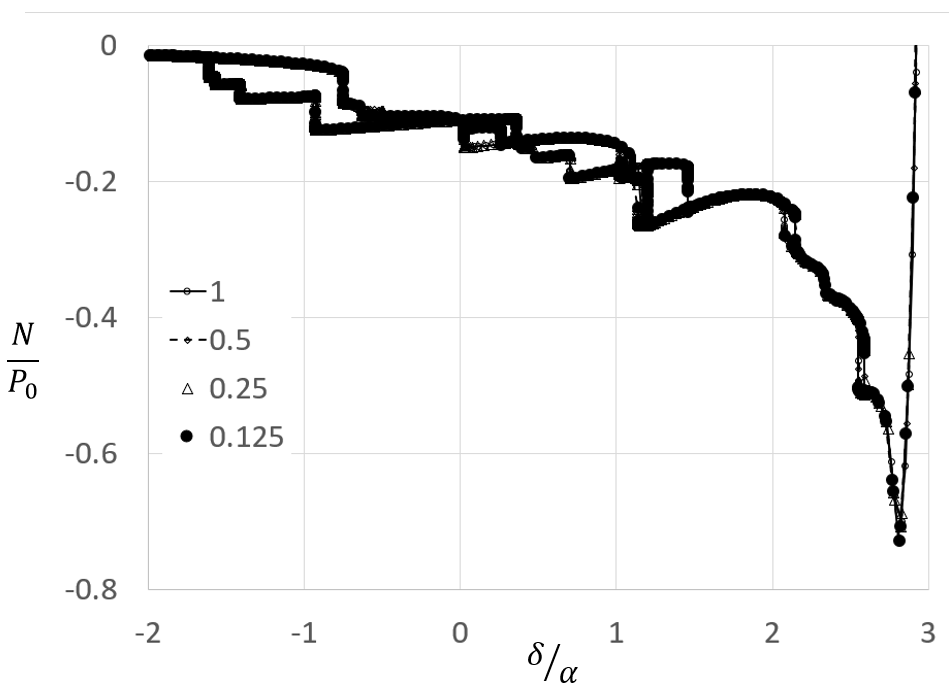


Figure S3 Normalized load vs Depth evaluated for different mesh sizes

**Intermittent fracture**

At contact state ‘2’, as shown in Fig. S6a, the gap along one end of the rough profile suddenly comes within the range of interaction. Along this span, as shown in Fig. S6b, the contact pressure distribution is rough with compressive and tensile stresses over small to intermediate length scales. Additionally, at the edge of this span, the contact pressure is tensile and has a crack tip type stress signature. At contact state ‘3’, as shown in Fig. S6a, there is an increase in the span of rough profile within the range of interaction. Accordingly, as seen in Fig. S6b, the crack tip type stress signature in the contact pressure distribution moves along the contact. Similarly, at contact state ‘4’, the rough profile at the other end comes within the range of interaction resulting in two crack tips like contact pressure distribution. These two ‘crack tips’ move towards each other to evolve the adhesive contact during approach. Hence, the evolution of adhesive contact during approach is like an intermittent fracture zipping process. During detachment of the contact, the process is similar to unzipping due intermittent fracture propagation.

Furthermore, as seen in Fig. 2, the contact area increases until state ‘5’ followed by decrease to state ‘8’. To understand this decrease in contact area, we have compared the contact stress distributions as shown in Fig. S7a. At contact state ‘5’, the pattern of compressive contact pressure has spikes closely spaced suggesting the contact on small to intermediate length scales. In comparison, the compressive contact pressure spike pattern is sparsely spaced at state ‘8’ suggesting the contact on large length scales. Therefore, the decrease in contact area from state ‘5’ to ‘8’ can be explained as the rearrangement of contact over small and intermediate length scales to large length scales. In addition, as shown in Fig. S7b, comparison of heights power spectrum at state ‘8’ of about 1% relative contact area with equivalent heights power spectrum of a non-adhesive contact shows the loss of self-affinity of an adhesive contact over several length scales. This is due to heights at smaller length scales being pulled up due to a large net attractive force on the surface. Beyond state ‘8’, the contacts on the large length scales are being pushed into net compressive load crossing the state of bonded contact at net zero load on the surface.


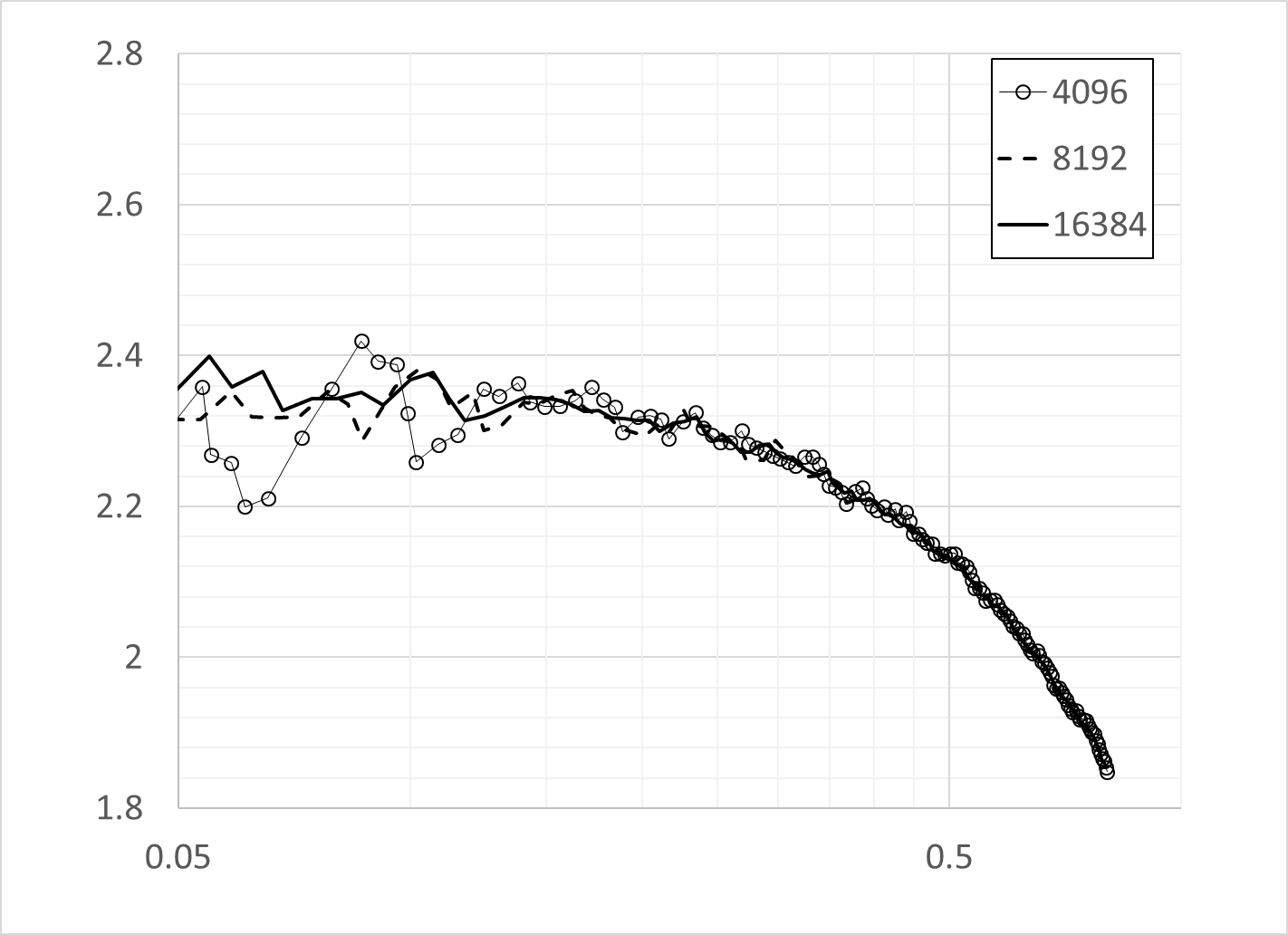

$$\boldsymbol{\kappa}$$

$$\frac{\boldsymbol{A}}{\boldsymbol{A}_{\boldsymbol{0}}}$$

Figure S4 Values of $\kappa=\frac{AE^{*}{h'}_{rms}}{N}$ for various system sizes ranging from 4096 to 16384 showing the proportionality of load with real contact area in the range of 5-8% for non-adhesive contact. The value of H used is 0.5.


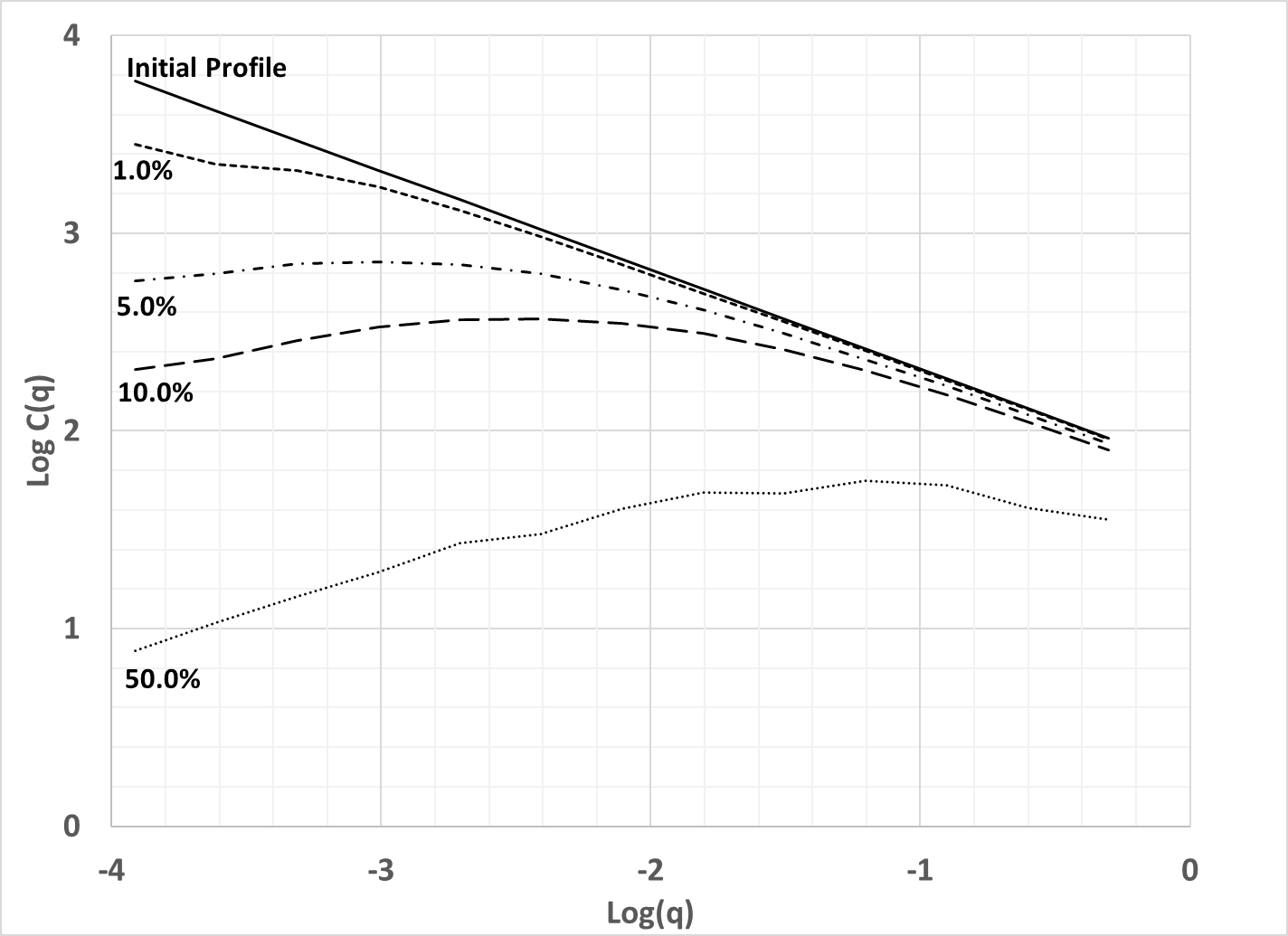


Figure S5 A comparison of power spectrum of heights at various contact area for non-adhesive contact. C(q) is the product of heights Fourier component h(q) and its complex conjugate h(q*), where q is the wave number


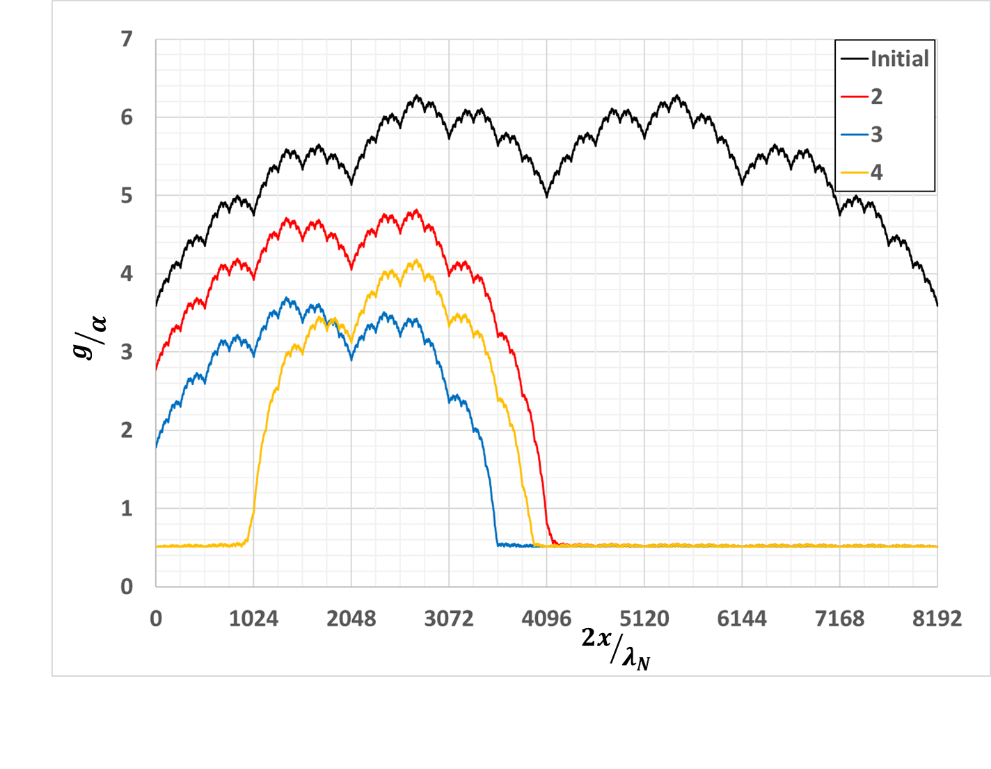


Figure S6. (a) A comparison of gap profile $g$ normalized with range of interaction $\alpha$ at contact states 2,3&4 (refer to Fig. 2) showing the evolution of contact through series of jump to contact instabilities.


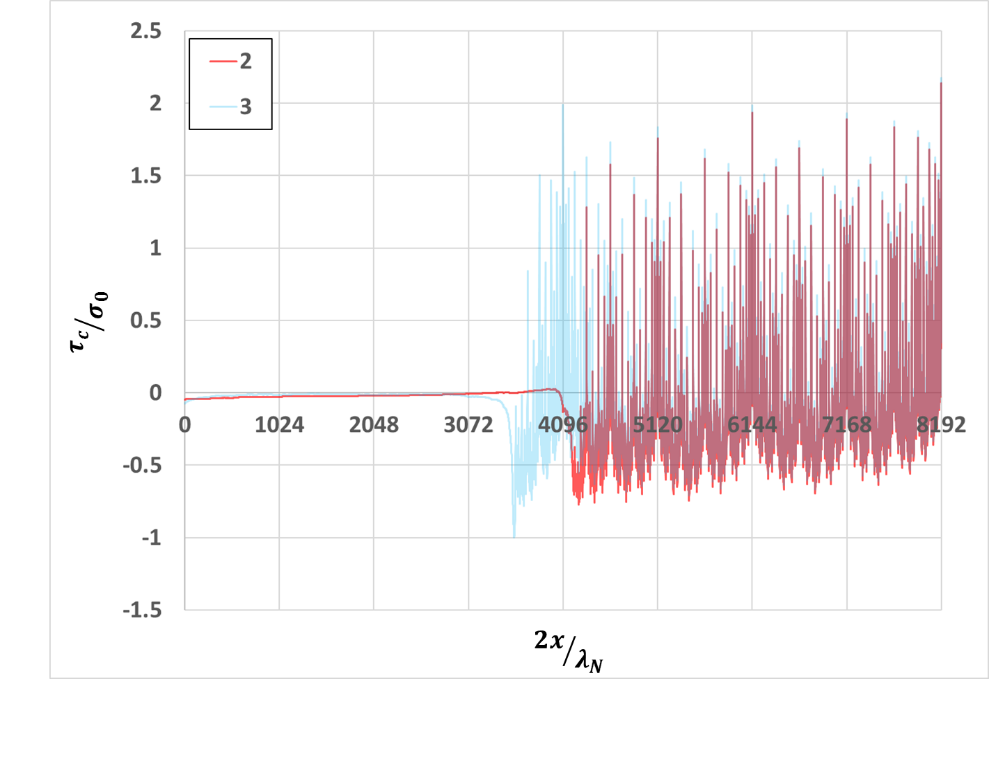


Figure S6. (b) A comparison of contact pressure $\tau_{c}$ normalized with maximum traction of LJ traction-separation law $\sigma_{0}$ at contact states 2&3 (refer to Fig. 2). The plot shows the jump to contact instability happening at small length scale and has crack tip like signature at the edge of contact.


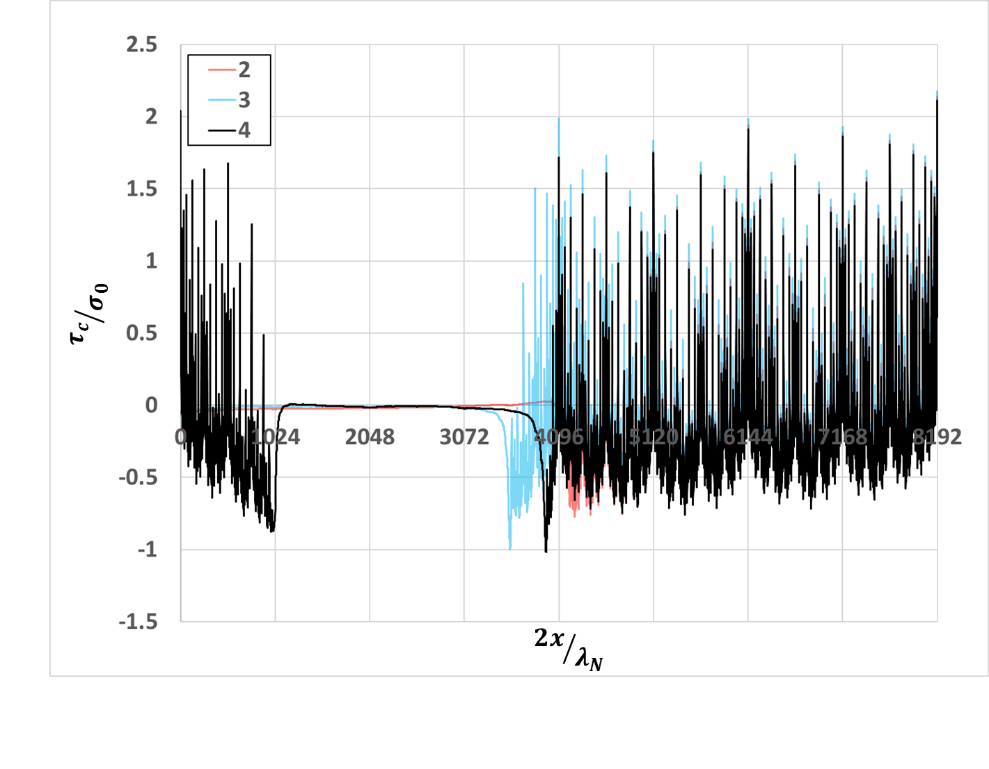


Figure S6. (c) A comparison of contact pressure $\tau_{c}$ normalized with maximum traction of LJ traction-separation law $\sigma_{0}$ at contact states 2,3&4 (refer to Fig. 2). The plot shows the evolution of adhesive contact is similar to a fracture zipping process.


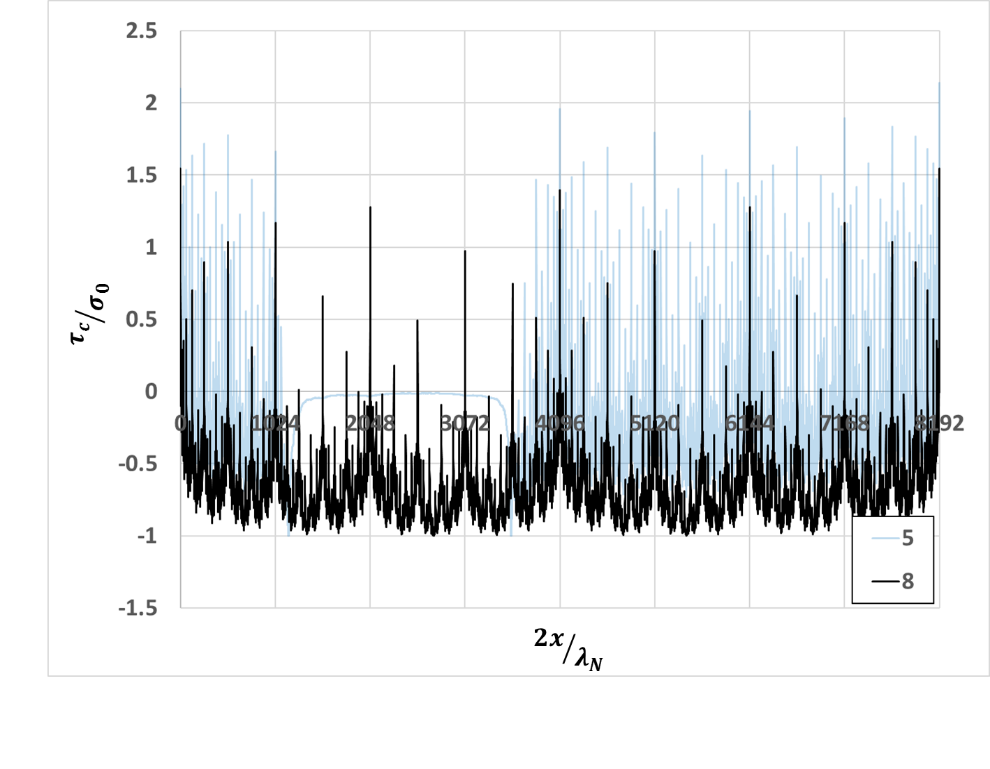


Figure S7. (a) A comparison of contact pressure $\tau_{c}$ normalized with maximum traction of LJ traction-separation law $\sigma_{0}$ at contact states 5&8 (refer to Fig. 2). The plot shows that the contact area evolution through jump to contact instability is happening at smaller length scales. At contact state 8 which has about 1% contact area, the contact area is redistributed over large length scales.


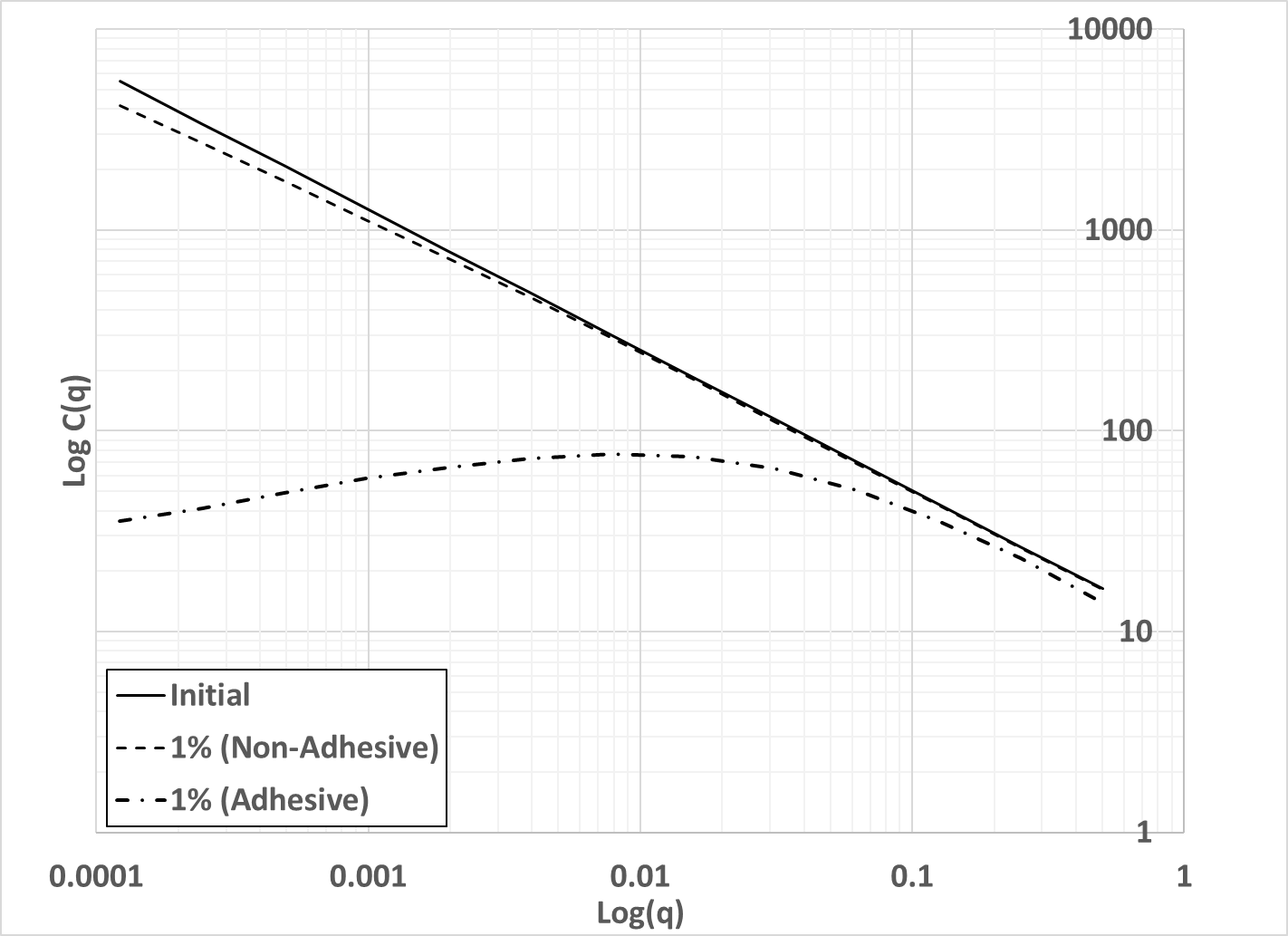


Figure S7. (b) A comparison of power spectrum of heights for 1% contact area with and without adhesion. The plot shows that the contact area for non-adhesive contact occurs at the large length scales and self-affinity is preserved for most for the smaller length scales. In contrast, even though, the contact is only of the large length scales, the heights profile looses its self-affinity for most of the intermediate length scales. C(q) is the product of heights Fourier component h(q) and its complex conjugate h(q*), where q is the wave number
